# Chloroplast DNA variation and phylogeography of *Eugenia uniflora L. (Myrtaceae)* in the Brazilian Atlantic forest

**DOI:** 10.1186/1753-6561-5-S7-P19

**Published:** 2011-09-13

**Authors:** Andreia Carina Turchetto-Zolet, Fabiano Salgueiro, Fernanda Cruz, Nicole Veto, Rogerio Margis

**Affiliations:** 1 Programa de Pós-Graduação em Genética e Biologia Molecular, Universidade Federal do Rio Grande do Sul (UFRGS), Brazil; 2 Departamento de Botânica, Universidade Federal do Estado do Rio de Janeiro (UNIRIO), Brazil; 3 Centro de Biotecnologia, Universidade Federal do Rio Grande do Sul (UFRGS), Brazil

## Introduction and objectives

The Atlantic Forest (AF) is considered the second largest tropical forest in South America with high species richness and endemisms, harboring a large diversity of animals, plants and habitats types [1]. This biome covers an area of more than one million square kilometres along the Brazilian coast and extending to eastern Paraguay and northeastern Argentina [2]. AF is considered one of the most threatened ecosystems on Earth due to intense disturbance, having been reduced to only 7.5% of its original area [1]. Despite an increase in research efforts in the past few years, studies of AF species diversification and knowledge about its evolutionary history is still scarce. Vegetation changes in the Atlantic Forest related to climatic changes during the Pleistocene have been registered in paleopalinological studies, with the replacement of large areas of forests by subtropical grasslands and savannas during cooler and drier conditions [3,4]. Also, studies of paleoclimatic models, predicted the presence of historically stable areas (refugia) in the Atlantic Forest during the Late Quaternary [5]. *Eugenia uniflora* L. (Myrtaceae), a shrubby tree with edible cherry-like fruits which is locally known as pitanga or Brazilian cherry. This species is one of the key species in the Atlantic rain forest geomorphological domain, which includes the Atlantic forest and the adjacent Restinga ecosystem [6]. *E. uniflora*occurs in areas of medium and large levels of rainfall and can also be found in different vegetation types and ecosystems. This species present economic and folk medicinal applications and is an important pioneer species in the Restinga ecosystem and has been used to recover and manage disturbed and fragmented areas. Our aim in this study was to investigate the phylogeography and genetic diversity of *Eugenia uniflora* to help elucidate the evolutionary history of this species as a model for gain insights into past vegetation patterns in the Brazilian Atlantic Forest.

## Methods

Forty-six populations of *E. uniflora* were sampled across the Brazilian Atlantic forest. The samples were collected as leaf material (silica gel dried) from natural populations. Total genomic DNA was isolated using the CTAB method [7]. Two cpDNA regions (*psb*A/*trn*H and *trn*C/*yc*F6) were using for population analysis based on sequence quality and degree of variation. These regions were amplified using universal primers. The individual consensus sequences were aligned through CLUSTALW [8] implemented in MEGA4 [9], then carefully improved manually. The genealogical relationships among haplotypes were estimated by using the median-joining method implemented in Network 4.2.0.1 (Fluxus Technology Ltd. at http://www.Fluxus-engineering.com). Molecular diversity estimates were calculated using Arlequin 3.1[10] and DNAsp 5.0 [11]. Genetic structure was further examined by the analysis of molecular variance (AMOVA), as implemented in Arlequin version 3.1.

## Results and conclusions

The total combined cpDNA matrix presented 1224 sites, which eight were variable. Eight haplotypes were found and the haplotype diversity (*h*) ranged from 0 to 0.733 and the nucleotide diversity (π) from 0 to 0.00140. Total haplotype and nucleotide diversities were 0.433 and 0.00088, respectively. The highest haplotype diversity was found in populations ITAP, PALM, CAPI and GRAV from the Atlantic forest South. Populations from Northeast were monomorphic. The most common haplotype was H1, present in 39 of 46 populations. The AMOVA analysis showed a very strong differentiation among all *E. uniflora* populations (F_ST_ =0.771, P<0.0001). These results can help a deeper understanding of the dynamics responsible for both ancient and more recent events that have shaped the current distribution of genetic variability in Atlantic Forest and also have implications for conservation efforts. For the long-term conservation of the genetic diversity of *E. uniflora*, it would be important to design strategies that aim to preserve most of its lineages. For such, the South region is a key piece, as it houses many divergent and endemic haplotypes and lineages.

Financial support: FAPERGS and CNPq
